# Right-sided ALPPS after preoperative emergency embolization of the right hepatic artery: case report with a favorable anatomy

**DOI:** 10.1093/jscr/rjad180

**Published:** 2023-04-13

**Authors:** Petras Laurinavicius, Philip C Müller, Soleen Ghafoor, Jan Philipp Jonas, Christian E Oberkofler, Pierre-Alain Clavien, Henrik Petrowsky

**Affiliations:** Department of Surgery, Swiss HPB and Transplantation Centre, University Hospital Zurich, Zurich, Switzerland; Department of Surgery, Swiss HPB and Transplantation Centre, University Hospital Zurich, Zurich, Switzerland; Institute of Diagnostic and Interventional Radiology, University Hospital Zurich, Zurich, Switzerland; Department of Surgery, Swiss HPB and Transplantation Centre, University Hospital Zurich, Zurich, Switzerland; Department of Surgery, Swiss HPB and Transplantation Centre, University Hospital Zurich, Zurich, Switzerland; Department of Surgery, Swiss HPB and Transplantation Centre, University Hospital Zurich, Zurich, Switzerland; Department of Surgery, Swiss HPB and Transplantation Centre, University Hospital Zurich, Zurich, Switzerland

**Keywords:** Liver surgery, Two-stage hepatectomy, Liver metastases, Colorectal cancer

## Abstract

In patients with extensive colorectal liver metastases (CRLM) and insufficient future liver remnant (FLR) a faster and more effective FLR augmentation than portal vein embolization is the associating liver partition and portal vein ligation in staged hepatectomy (ALPPS). Before ALPPS, the presence of arterial blood supply to the subsequently resected hemiliver must be ensured. We present a case with neoadjuvant-treated CRLM and insufficient FLR who developed a large intrahepatic hematoma after liver biopsy. For continuous bleeding, the right hepatic artery was embolized. Fortunately, an accessory right hepatic artery arising from the superior mesenteric artery was present, which enabled the ALPPS procedure to be performed. After ALPPS, the patient did not experience liver failure. The case exemplifies that preoperative evaluation of the vascular supply of the liver is of paramount importance in advanced hepatic surgery such as ALPPS.

## INTRODUCTION

In patients with extensive colorectal liver metastases (CRLM) and insufficient future liver remnant (FLR), a two-stage approach may become necessary to achieve safe and curative resection. Current gold standard for such a two-stage procedure is portal vein embolization (PVE) followed by hepatectomy 6–8 weeks later after sufficient FLR augmentation [1]. Another way to achieve a faster and more effective FLR augmentation than PVE is the associating liver partition and portal vein ligation in staged hepatectomy (ALPPS) [2]. In a first step, ALPPS usually consists of cleaning the FLR, portal vein ligation and (partial) parenchymal transection leaving only the hepatic arterial blood supply for the future resection side. This ensures increased portal blood flow to the healthy hemiliver inducing hypertrophy and facilitating resection of the contralateral side within 7–10 days in a second step. The main indication for this procedure is CLRM, to a lesser extent hepatocellular carcinoma (HCC), or intrahepatic cholangiocarcinoma [3]. Before considering ALPPS, the presence of arterial blood supply to the subsequently resected hemiliver must be ensured [4].

## CASE REPORT

**Figure 1:**
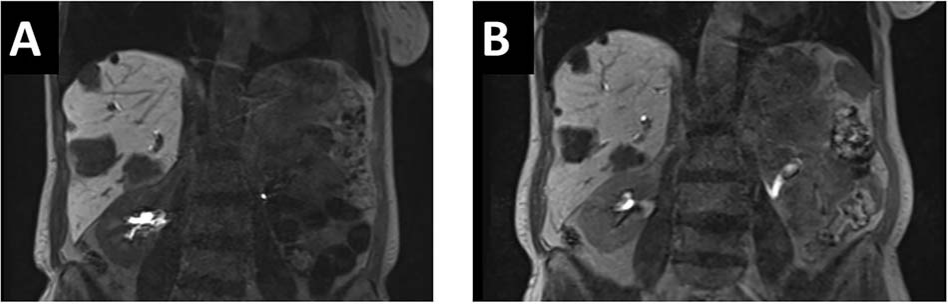
(**A**). In the initial pre-chemo MRI of the liver multiple liver metastases in all liver segments could been seen. The largest were in segments II/III (26 mm), V (37 mm) and segment VI (40 mm). (**B**) After 12 cycles of chemotherapy a size reduction of liver metastasis up to 12% could be achieved. The metastases in liver segments V and VI decreased from 43 to 38 mm and from 37 to 33 mm.

A 65-year-old, otherwise healthy patient was diagnosed with a moderately differentiated adenocarcinoma of the sigmoid colon with a KRAS mutation. Laboratory results showed an elevated CEA of 69 ug/l. Staging of the liver showed multiple liver metastases in all segments with 10 lesions in the right and 8 in the left hemiliver (Fig. 1A).

**Figure 2:**
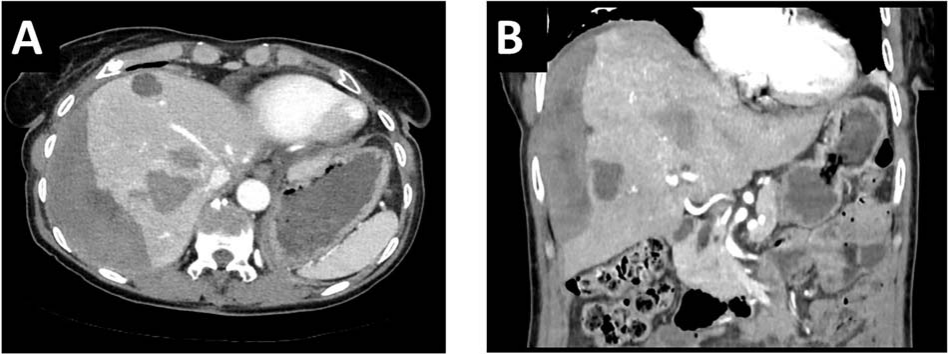
(**A**/**B**) CT-scan showing a large intrahepatic hematoma after liver biopsy on the right side.

**Figure 3:**
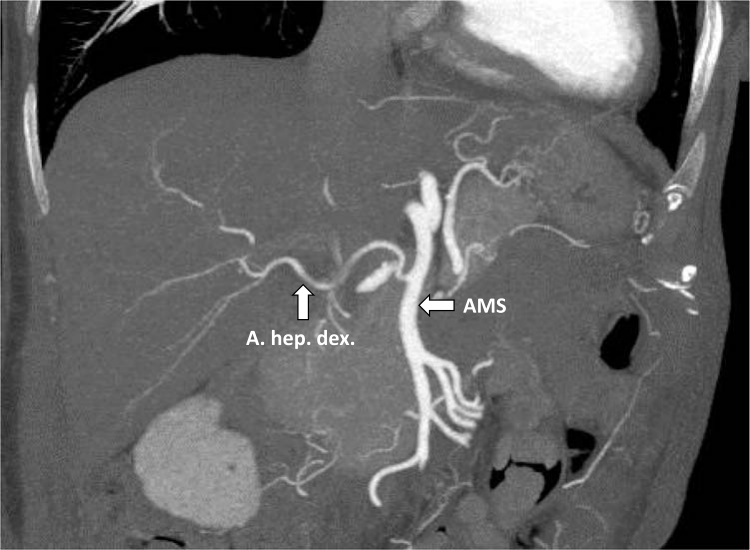
CT-angiography showing the accessory right hepatic artery originating from the superior mesenteric artery.

After multidisciplinary tumor board (MDT) discussion, neoadjuvant chemotherapy with six cycles of FOLFOXIRI and Bevacizumab followed by liver-first approach was decided. Restaging after 3 months showed stable disease of the CRLM, therefore, chemotherapy was continued. After 12 cycles, restaging revealed a size reduction of 12% of the CRLM (Fig. 1B). CEA decreased from 69 to 6.1 ug/l. The patient was then admitted to our department for the evaluation of a curative intent CRLM resection. The radiological work-up revealed three remaining metastases on the left- and seven on the right hemiliver. Volumetry illustrated a standardized FLR (sFLR) for a right hemihepatectomy of 30%. In an MDT and the patient was deemed amenable to ALPPS surgery due to the marginal sFLR, bilobar metastases and exceptionally good performance status. Preoperative liver function tests showed normal liver function (LiMAx 450 mcg/kg/h, cut-off >315 mcg/kg/h and ICG R15: 4.0%, cutoff <12%). Although liver biopsy is not routinely performed before ALPPS, MDT recommended a biopsy of the healthy liver to rule out chemotherapy-associated steatohepatitis after 12 cycles of FOLFOXIRI and Bevacizumab in order to ensure a safe liver resection. The biopsy showed signs of subacute hepatocyte demise without fibrosis or cirrhosis. The day after the liver biopsy, the patient collapsed and was admitted to the emergency department of another hospital. CT scan showed a large intrahepatic hematoma (Fig. 2) without active bleeding probably secondary to the biopsy procedure. Due to a hemoglobin drop of 12 g/l a day later and the enlargement of the hematoma in follow-up CT scan, the patient was admitted to angiography, which showed several subcapsular liver bleedings, therefore the right hepatic artery was embolized. Luckily, an accessory right hepatic artery arising from the superior mesenteric artery was present. This enabled us to stay on track with the ALPPS procedure as the right hemiliver would be otherwise without any blood supply (Fig. 3). In synopsis of those findings, the operation was scheduled.

**Figure 4:**
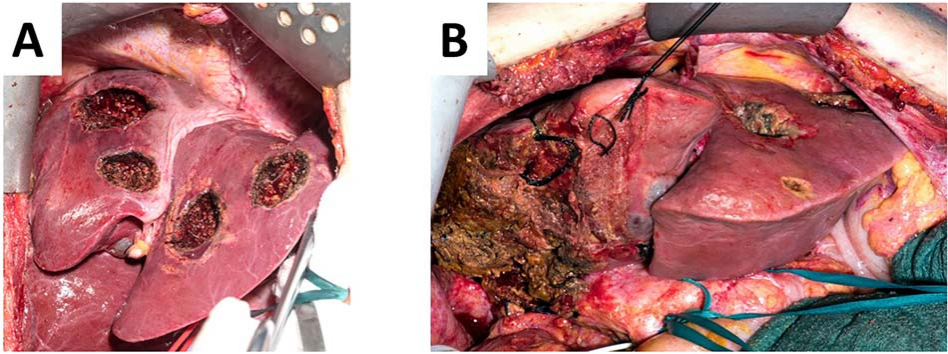
(**A**). Left hemiliver after cleaning of the FLR with five atypical liver resections. (**B**) Remaining left hemiliver after completion right hemihepatectomy during ALPPS step 2.

During ALPPS step 1, the right accessory artery was clearly identified. Intraoperative ultrasound revealed five metastases on the left side, which were all removed by atypical resections (Fig. 4A). Since the biopsy-induced liver hematoma on the right side was intrahepatic, hematoma evacuation was avoided during ALPPS step 1 due to the potential risk of liver injury or mobilization-associated bleeding. In the follow-up volumetry 6 days after ALPPS step 1, the sFLR increased from 30 to 59%. Liver function tests after step 1 showed sufficient hepatic function (LiMAx 326 mcg/kg/h and ICG R15 2.7%). Therefore, ALPPS step 2 including evacuation of the perihepatic hematoma and completion right hemihepatectomy was performed 7 days after step 1 (Fig. 4B). In the postoperative course, there were no signs of liver failure. However, the patient developed a wound infection (Clavien-Dindo grade II), which was managed by bedside vacuum-assisted closure therapy. The patient was discharged on postoperative day 10 following ALPPS step 2 surgery. Adjuvant chemotherapy with FOLFOX for 3 months was recommended. The primary tumor in the sigmoid colon regressed during chemotherapy and robotic-assisted left hemicolectomy was performed 6 months after the ALPPS procedure. At last follow-up, 15 months after ALPPS there were no signs of tumor recurrence.

## DISCUSSION

At presentation, ~75% of patients with CRLM have unresectable disease mainly due to extensive tumor burden and insufficient FLR [5]. ALPPS can show impressive volumetric liver growth and CLRM are considered the main indication for this highly specialized procedure [6]. Although there is not much oncological outcome data for this procedure, first studies show pleasing long-term oncologic results [7].

Our presented case is the first to describe a successful ALPPS procedure after an emergency embolization of the right hepatic artery due to active bleeding thanks to the anatomical variation with an accessory right liver artery. In general, embolization of right hepatic artery is an absolute contraindication for right-sided ALPPS as ligating of the right portal vein would have left the right liver without any blood supply. Alternative options for this patient would have been a conventional two-staged hepatectomy after PVE or liver venous deprivation (LVD), as LVD induces a greater and faster liver hypertrophy than PVE. However, these methods were shown to be inferior to ALPPS in terms of liver hypertrophy rate and successful resection rates of CLRM [8]. Fortunately, in our case, arterial blood flow after ALPPS step 1 could be ensured through an accessory right liver artery, which was spared from the emergent embolization procedure. Therefore, ALPPS procedure could be performed as planned. A previous case report [9] described a patient with an aberrant right hepatic artery arising from the superior mesenteric artery (type III according to the Michel classification [10]), which was preserved during first stage of ALPPS. However, on the second postoperative day, the patient from the report by Sanjeevi *et al*. developed laboratory signs of an acute liver failure due to occlusion of right hepatic artery. Nevertheless, the FLR increased from 290 to 746 ml within 6 days. Despite the occluded right artery, the patient underwent stage 2 on the seventh postoperative day and had an unremarkable further recovery [9]. In previous unhealthy livers, the increase of the FLR may be limited due to severe fibrosis or cirrhosis. In patients who failed to achieve adequate FLR growth after stage 1, Peng *et al*. described an ALPPS modification in which the arterial supply of HCC was occluded using transarterial embolization (TAE) 14 days after ALPPS stage 1, followed by ALPPS stage 2 7 days later [11]. In this relation, TAE-associated complications were grade A posthepatectomy liver failure, tumor lysis syndrome and ascites resulting in a TAE-specific morbidity rate of 20%. Nonetheless, TAE resulted in significant increases of FLR, kinetic growth rates from 8.9 to 19.5 ml/day after TAE, showing a promising modification of ALPPS in HCC.

## CONCLUSION

For successful ALPPS, adequate arterial blood supply to the resected hemiliver needs to be preoperatively ensured. Luckily, the anatomy of an accessory right hepatic artery allowed us to perform ALPPS after emergency embolization of the right hepatic artery. In addition, the case exemplifies that preoperative evaluation of the vascular supply of the liver is of paramount importance in advanced hepatic surgery such as ALPPS.

## Data Availability

Data available on request from the authors.
